# A Wireless and Passive Low-Pressure Sensor

**DOI:** 10.3390/s140203065

**Published:** 2014-02-17

**Authors:** Pascal Nicolay, Martin Lenzhofer

**Affiliations:** Carinthian Tech Research (CTR AG), Villach/St. Magdalen 9524, Austria; E-Mail: martin.lenzhofer@ctr.at

**Keywords:** low pressure sensor, Pirani, wireless, passive, SAW, inductive heating

## Abstract

This paper will discuss the results obtained with a first prototype of a completely passive and wireless low pressure sensor. The device is a heat conductivity gauge, based on a wireless and passive SAW temperature sensor. The required heating energy is applied to the sensor using inductive coupling. The prototype was successfully tested in a vacuum chamber. Its equilibrium temperature changed drastically and in a reproducible way when pressure steps were applied. However, the response time was very long. A model is provided to account for the sensor's behavior. It is then used to show that the response time could be strongly improved using basic design improvements. Further possible improvements are discussed.

## 1. Introduction

Low pressure sensors of the Pirani kind are routinely used, most notably in the semiconductor industry. They are robust sensors with a simple working principle. A constantly heated element is plunged into a rarefied gas and the pressure is deduced from its equilibrium temperature. Indeed, the equilibrium temperature depends on the gas thermal conductivity which itself depends on the gas pressure, especially at low pressure where the dependence is quasi linear. Another operating mode consists in adjusting the heating power to keep the sensor's temperature constant and then deducing the pressure from the heating power itself. This is the preferred mode of operation for standard Pirani gauges. In these commercial gauges, the sensing element is a hot wire. Its temperature is deduced from its resistance. The wire being a long and thin cylinder, its surface/volume ratio is big. Hence, gas-surface interaction is enhanced and thermal inertia is reduced. This basically means a very short time to equilibrium, or response-time. Besides, the wire is mounted in a Wheatstone bridge which allows for very quick and very sensitive detection of resistance and therefore temperature imbalance. The bridge drives the power source through a feedback-loop. This enables the almost instantaneous adjustment of the heating power to re-balance the bridge and therefore keep the wire at a constant temperature. Thanks to this clever configuration, standard Pirani gauges have response times shorter than 20 ms for a measurement uncertainty of about 15% over a wide pressure range, from 0.05 Pa (5.10^−4^ mbar) to atmospheric pressure. They are often the best choice for reliable and durable pressure monitoring under medium vacuum, between 0.05 Pa and 100 Pa (1 mbar).

However, a standard Pirani gauge costs around 1.5 k€, it is quite big (the standard housing is the size of a small coffee mug) and it comes with an electronic reader which is required to extract the pressure information. It is therefore difficult if not impossible to mount a Pirani gauge directly *inside* a small vacuum chamber, let alone vacuum housings for special devices like MEMS sensors. To answer the demand for small and accurate Pirani sensors, a lot of efforts have been dedicated worldwide to the development of MEMS Pirani gauges [1–3] and the first solutions are already commercialized [4,5].

Nevertheless, all of these solutions are essentially miniaturized versions of the standard Pirani gauge. The operating principle is identical. This means that the sensors have to be wired to their conditioning electronics, and the need for active electronics next to the sensor is already a big enough constraint to make the existing MEMS Pirani gauges a non-viable solution for a whole range of possible applications where small, embedded, low-cost, accurate and maintenance-free “patch” sensors would be needed. In this case, the ideal solution would be a passive and wireless Pirani device. To realize such a sensor, only two functions would actually have to be implemented in a passive and wireless way: heating and temperature monitoring. For instance, a hot wire connected to a flat spiral coil designed for efficient energy transfer via inductive coupling would already constitutes a reasonably good passive and wireless low-pressure sensor. The “patch” made of the wire and the coil would be mounted inside a vacuum chamber. Provided that the vacuum chamber has a glass window, heating energy would be transferred to the coil using inductive coupling. This energy would be dissipated in the hot wire, making its temperature change. And the pressure-dependent temperature would then be monitored from outside using an IR-thermometer. If possible in principle, this solution would be quite hard to implement and use. First of all, the accuracy of the IR-thermometer would not be good enough to allow for an accurate determination of the inner pressure. In addition, IR-thermometer would be easily blinded by hot gases flowing around the sensor. To solve these two issues, one could rely on another “more robust” frequency range to transmit the temperature information and use a more accurate measuring principle. One possible solution would be to use a Surface Acoustic Wave (SAW) temperature sensor instead of the hot wire. SAW sensors are small, robust, passive and wireless. An extensive literature exists on the working principles and possible applications of SAW wireless sensors [6,7], notably at high temperature [8–10]. Off-the-shelf SAW sensors can operate at 2.45 GHz and withstand temperatures up to 300 °C for thousands of hours. The temperature accuracy achieved in wireless and passive mode is better than ±0.1 °C even in aggressive and perturbed environment [11]. Therefore, a heated SAW temperature sensor could certainly constitute a good wireless and passive low-pressure sensor. The additional elements that would be needed here are (again) a coil for wireless energy transfer, a resistive heater connected to the coil and attached to the SAW device, and an antenna for remote interrogation of the SAW device (for temperature monitoring *vs.* pressure).

The idea of a “SAW Pirani” is not new. The principle was invented in 2006 [12] and successfully tested in the following years [13]. The sensitivity was proven to be very high, but the response time was poor. Efforts have been made to improve the response time by reducing the sensor's dimensions [14] or improving the sensor's *modus operandi* [15,16], but a really efficient and practical solution still remains to be found. It is now clear that the development of a SAW Pirani that would be at the same time very fast (*i.e.*, very small) and accurate-enough to compete with standard and MEMS-based Pirani gauges would require advanced packaging and hetero-integration techniques. Unfortunately, this would most certainly result in an overpriced and unsellable device. However, a wireless and passive version of the SAW Pirani (WiPirani), would present numerous advantages over competing solutions that might open new interesting perspectives in the field of low pressure monitoring, where only small, cheap and robust embedded sensors can be used.

We report here the experimental results obtained with the first prototype of a WiPirani sensor (Section 2). The prototype was used to monitor the inner pressure of a fused quartz vacuum tube, from outside the tube and without any form of embedded electronics. High pressure sensitivity was observed, even at low operating temperature. The response-time was poor but could be strongly improved through redesign and miniaturization. To prove this point, a complete numerical model of the sensor is provided in Section 3. The coils used for inductive heating are simulated using Finite Element Modeling Magnetics (FEMM). A thermal/electrical analogy is used to simulate the behavior of the sensor itself using Quite Universal Circuit Simulator (QUCS). A good agreement is observed between simulated and experimental data. This validates the model which is then used to check and confirm the positive effect of some basic design improvements on the response time. The next development steps are shortly described before conclusion.

## 2. Experimental Section

**Figure 1.**
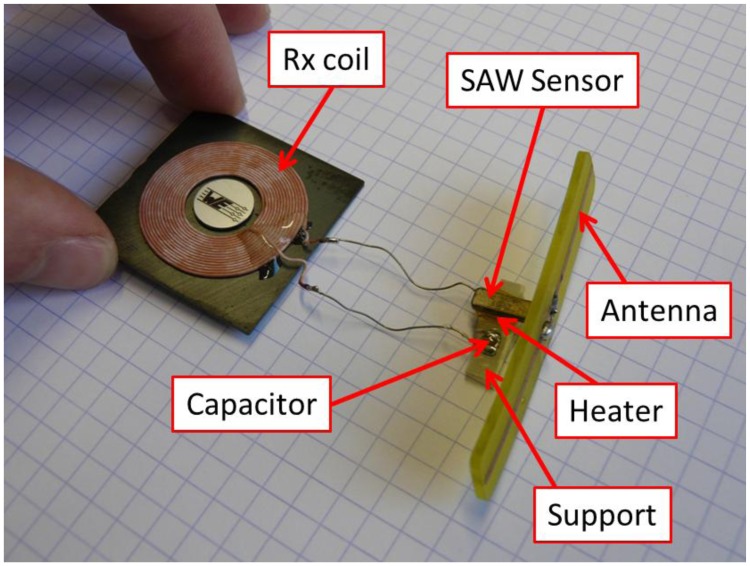
The WiPirani prototype, made of a SAW Tag with its antenna, a planar coil designed for inductive power transfer and a heating resistor. The resistor is located between the SAW Tag and its PCB support. It is not visible on the picture. The underlying grid is made of 0.5 mm × 0.5 mm squares.

The sensor prototype is presented in Figure 1. The two main components are a planar spiral coil and a SAW Tag. The coil is one of the two coils used in the wireless power charging device developed by Texas Instruments (WE-WPCC, distributed by Würth Elektronik GmbH & Co. KG, Germany). We used the receiver (Rx) coil, which is smaller. The SAW Tag was designed by CTR AG and already used for several applications in different fields, ranging from biomedical to automotive. A detailed description of its working principle can be found elsewhere [17]. The SAW Tag responds to a radar-like excitation with a series of echoes. The relative delays between the echoes depend on the temperature. This commonly allows for remotely sensing the temperature up to 5 m in air, with a standard accuracy of 0.1 °C. The CTR SAW Tag we used operates at 2.45 GHz and comes in Kovar housing. It can withstand and work at relatively high temperature (200 °C) for a long period of time (>1,000 h). Its dimensions are 10 mm × 3.1 mm × 1.8 mm. In the WiPirani prototype, the SAW Tag was equipped with an in-house manufactured dipole antenna and with a small heating resistor (35 Ω). The heating resistor was glued on the SAW Tag's bottom surface and electrically connected to the coil. Then, the SAW Tag was glued on a small PCB. Consequently, the heating resistor was located between the PCB and the SAW Tag and was not visible anymore. The remote and passive monitoring of the Tag temperature was performed using a classical CTR FMCW SAW Reader. Its working principle was described elsewhere [18].

**Figure 2.**
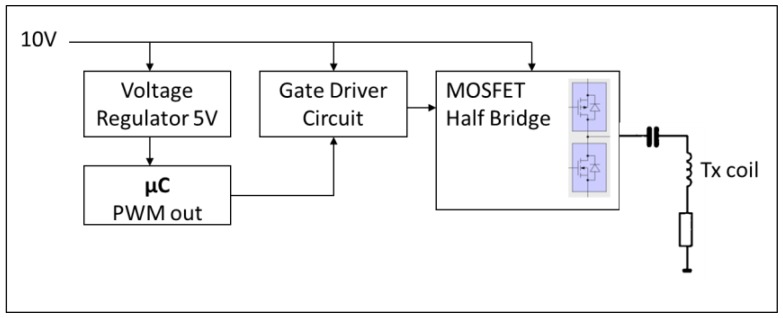
Bloc diagram of the driving electronics of the wireless heating stage.

Non-contact inductive heating was used to heat up the SAW Tag with a constant and well-controlled heating power. To transmit the energy, a second identical spiral coil was used. The driving electronics of this transmitter (Tx) coil was designed at CTR for similar purposes (*i.e.*, wireless energy transfer) and advantageously used for the present feasibility study. It consists essentially of a microcontroller, a switch or gate driver and a MOSFET-based half bridge to adjust the frequency, the duty cycle and the power level. The device is supplied with a voltage of 10 V. The output square wave AC signal was fed into the resonance circuit formed by the coil and the additional capacitor. The capacitor was used to make the whole circuit resonate at 125 kHz. In our case, a capacitor of 0.1 μF was used to match the coil which presents an inductance of 10 μH at 125 kHz. The bloc diagram of the electronics is presented in Figure 2.

**Figure 3.**
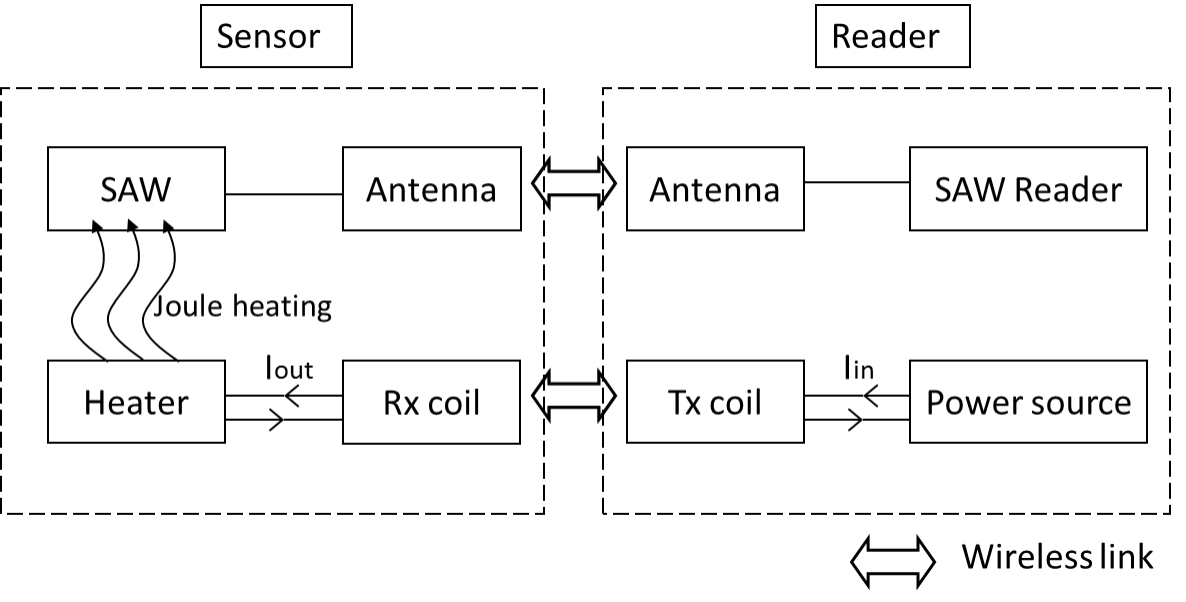
Schematics of the whole WiPirani system, including the WiPirani sensor and its reader.

Schematics of the complete system are presented in Figure 3. It is stressed that CTR already developed a handheld version of its FMCW reader, for specific purposes. The inductive heating electronics presented in Figure 2 could be easily integrated in the handheld device to form a unique, easy-to-handle interrogation device.

**Figure 4.**
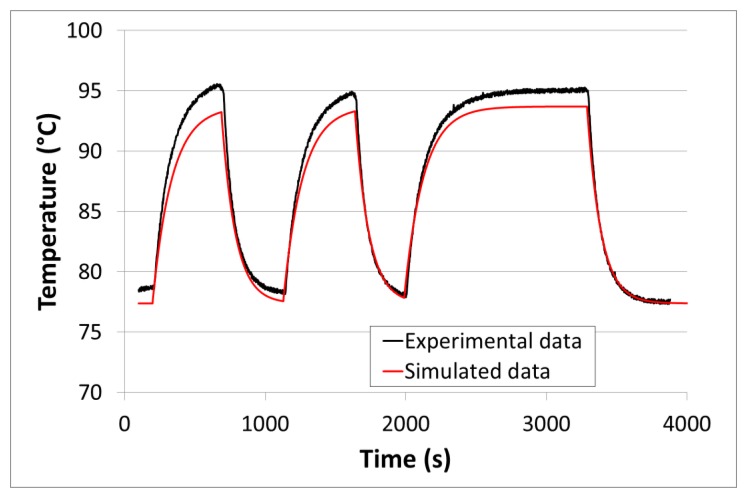
Experimental and simulated temperature behavior of the first WiPirani demonstrator submitted to pressure cycles, at low pressure. The pressure was changed between two levels–100 Pa and 0.1 Pa–using an on/off valve. The pressure changed quickly between these two levels (the pressure equilibrium was reached in a few seconds only).

For test purposes, the sensor was placed into the vacuum tube of a high-temperature tube furnace. The tube is made of fused quartz. It has a thickness of 2.5 mm. It is connected to “house” vacuum (100 Pa) and to an additional pumping circuit driven by a turbo molecular pump. A reference pressure gauge is also connected to the tube. During the experiments, room temperature was 29 °C. The Tx coil was mounted outside the tube. The distance between the Rx and Tx coils was close to 15 mm. The reading antenna was placed outside the tube at a distance of about 15 cm. An (inductive) heating power of about 1.5 W was used all along the experiment. After the heating power was switched on, “house” vacuum was first applied inside the tube. The pressure quickly stabilized around 100 Pa. After the sensor reached its equilibrium temperature, a valve connecting the tube to the high-vacuum pumping circuit was turned on and off several times. This generated fast pressure steps in the tube, between 100 Pa and 0.1 Pa. The initial equilibrium temperature before stepping was around 80 °C. This temperature will be referred to as the ‘working temperature’ in the following chapters. The temperature evolution *versus* time is showed in Figure 4.

It can be seen that the pressure steps made the temperature change drastically, in a reproducible way. An equilibrium temperature of 95 °C was reached under high vacuum (0.1 Pa) *versus* 78 °C at 100 Pa. This means a sensitivity of 0.17 °C/Pa, below 100 Pa. This demonstrates the high pressure sensitivity of the WiPirani system, in the low pressure range at least. With a reasonable resolution of 0.1 °C, such a system could easily detect pressure variations of 0.6 Pa (6.10^−3^ mbar). It is worth noting that the resolution could be significantly improved by operating at higher temperature [13]. This opens interesting perspectives that will have to be further investigated in future work.

However, the prototype also shows obvious drawbacks. One limitation is the huge response time observed during the experiments (τ > 5 min). Such a sensor could still be used for some specific applications where a fast response-time is not required. This is the case for applications where good vacuum must be guaranteed over long periods of time and only vacuum default has to be detected, most often during maintenance operation. However, such a long response time would strongly restrict the range of possible applications for the sensor.

As will be shown in Section 3, the huge response time was due to the big size of the prototype's building blocks, especially the bulky antenna used for the interrogation of the SAW sensor. The big size of these components implies a big thermal inertia and therefore a long response time. Ways to strongly improve the response time will be discussed in Section 3. We believe that the response time can be made smaller than a few seconds, especially if MEMS technology is employed.

The second limitation is a more fundamental one. It is linked to the working principle itself. Indeed, the temperature depends on the pressure but also on the distance between the Rx and Tx coils. A small change in the respective position of the inductive coils would change the amount of transmitted energy and have a non-negligible effect on the sensor's temperature and therefore on the pressure reading. To allow for a practical and easy use of the whole system, a technical solution to this problem will have to be found. This issue will be addressed in future work.

## 3. Modeling and Discussion

In the following section, we first describe a complete model of the prototype. The wireless power transmitter device (*i.e.*, the two coupled coils) was modeled using FEMM, a finite element modeling freeware dedicated to the simulation of magnetic effects [19] (see Section 3.1). The thermal behavior of the WiPirani was then computed using QUCS, a well-known circuit simulator, after conversion of the prototype into its equivalent circuit (see Section 3.2). A simple, easy-to-build equivalent circuit could be used here because thermal radiation and convection can both be neglected as a first approximation. Indeed, in the experimental configuration described above, thermal radiation accounted for less than 15% of the outgoing energy flow and convection couldn't take place at all, at low pressure. Hence, the only phenomenon to be taken into account is thermal conduction which can be easily modeled using equivalent resistors (analogous to thermal resistance) and capacitors (analogous to thermal capacity). The results showed good agreement between model and experiment. In Section 3.3, the model is used to predict what could be the response time of a slightly improved version of the WiPirani prototype that is a WiPirani equipped with a wire antenna.

### 3.1. Wireless Heating Model

**Figure 5.**
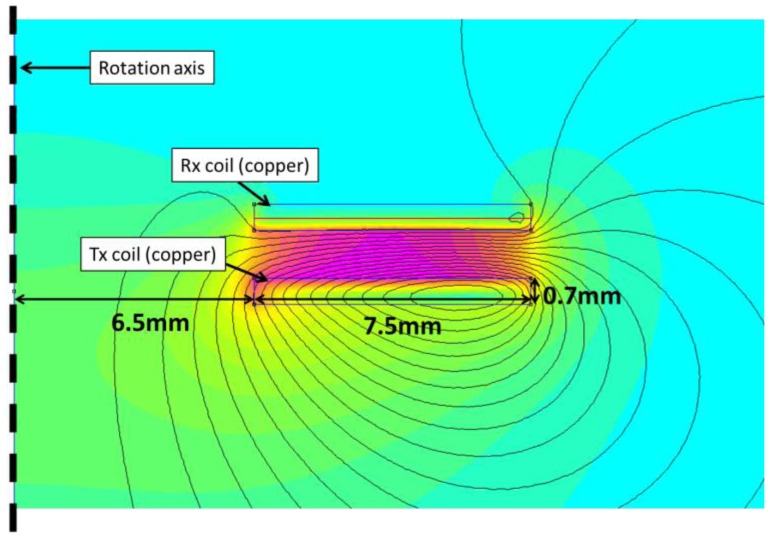
FEMM Model of the power transmission component of the WiPirani.

The power transmitting coils were modeled using FEMM. The layout is presented in Figure 5. The solution region was chosen to be a sphere with a radius of 40 mm. The Tx coil was positioned on the equatorial plane. The sphere was filled with air. Asymptotic boundary conditions were applied on the outer boundaries. The individual windings were not considered and each coil was approximated as a flat copper disc with a circular hole in its center.

**Figure 6.**
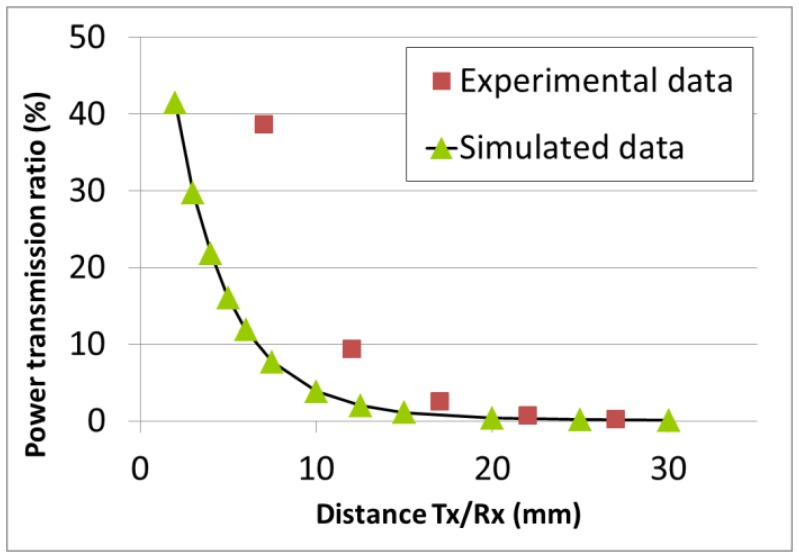
Power transmission ratio *vs.* distance.

The ferrite core and ferrite support were not considered. Indeed, we observed–using FEMM models including these two elements–that they have almost no effect on the power transmission ratio. A current of 1 A was injected in the Tx coil, resulting in a current density of 0.19 MA/m^2^. The AC frequency was 125 kHz. The induced magnetic field is also shown in Figure 5 (field lines and field density). The induced current was computed in the Rx coil, along the center line marked in red. As a very first approximation, the average value along the line was considered to be the average induced current in the Rx coil. The computation was performed for different Rx/Tx distances (center to center), ranging from 2 mm to 30 mm. The power transmission ratio was given by the squared ratio between the induced and injected current densities (the respective impedances of the Rx and Tx coils were considered equal). The power transmission ratio is plotted in Figure 6. Additionally, the two coils were characterized experimentally. The Tx coil was fixed in front of the Rx coil, which was itself mounted on a sliding rail. This time, a constant voltage (U_in_) of 15 V was injected in the Tx coil. The induced voltage (U_out_) in the Rx coil was recorded as the Rx/Tx distance was increased. The respective impedances of the Rx and Tx coils were again considered equal and the power transmission ratio was given by (U_in_/U_out_)^2^. It is also plotted in Figure 6. There is a reasonably good agreement between experimental and simulated results above 15 mm. The experimental power transmission ratio is actually better than the computed one. The rough approximations used to compute the induced current as well as the additional capacitance used to generate a resonance at 125 kHz and therefore improve the transmission efficiency might account for the observed discrepancy, which strongly increases at short distances. However, the agreement is already good enough to validate the model as a first approximation and allow for its use as a simple design tool if the Rx/Tx distance is bigger than 15 mm.

### 3.2. Thermal Behavior Model

**Figure 7.**
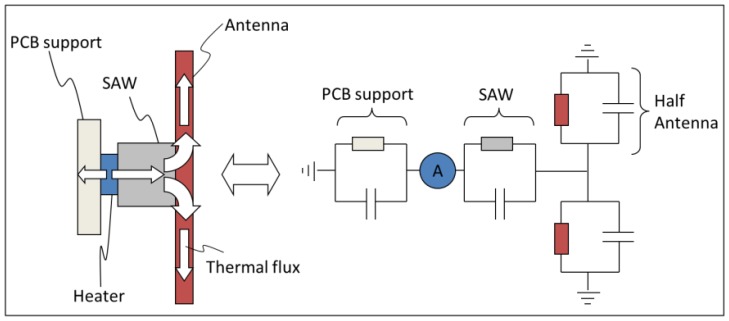
Thermal-Electrical conversion.

**Figure 8.**
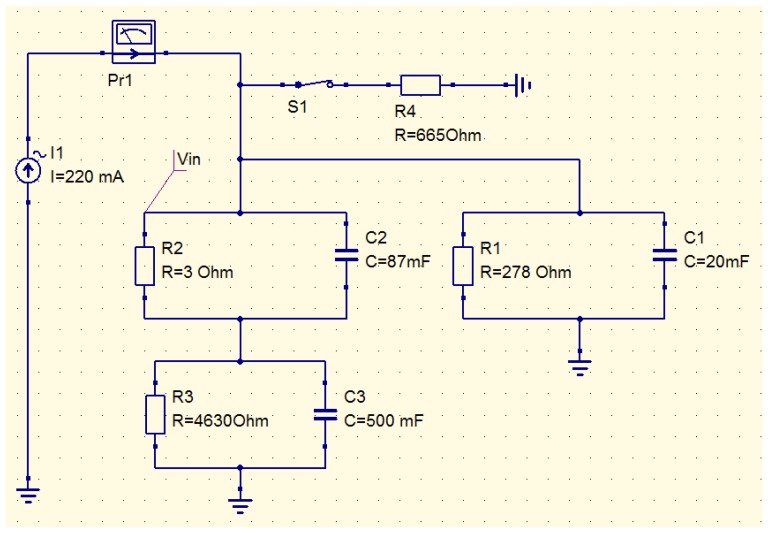
QUCS model of the WiPirani prototype.

As already mentioned above, it is possible to use a simple circuit simulator to compute the thermal behavior of the WiPirani. The main step consists in converting the physical layout into its equivalent RC circuit, using a simple thermal-electrical analogy. The principle of the conversion we made is described in Figure 7. To calculate the equivalent resistance and capacitance of the different elements, the only parameters we used were the thermal conductivity, specific heat capacity, density and geometrical dimensions of these elements. The analogy between Ohm's law for electrical circuits and the Fourier's law of heat conduction and the analogy between the electrical and thermal capacitances yield the following relationships. The equivalent capacitance is directly equal to the heat capacity, one farad (F) corresponding to one J/K. The equivalent resistance is directly equal to the thermal resistance, one ohm (Ω) corresponding to one K/W. For one half of the PCB micro-strip antenna (made of FR4 with a thermal conductivity λ of 0.3 W·K^−1^·m^−1^, a density ρ of 1,850 kg/m^3^, a specific heat capacity C_v_ of 600 J·kg^−1^·K^−1^, a length L of 25 × 10^−3^ m and a cross-section S of 9 × 10^−6^ m^2^) we obtain the thermal resistance R_ant_ = (1/λ)·(L/S) ∼9,260 Ω and the thermal capacity C = ρ·L·S·C_v_ ∼250 mF. In the equivalent thermal circuit, the antenna is made of two half antennas ‘in parallel’ because the SAW sensor is physically connected to the middle of the dipole antenna. Therefore, using electrical transformation laws for parallel circuits, it can be represented by one single circuit with R_ant_ = 4,630 Ω and C_ant_ = 500 mF. The equivalent resistance and capacitance of the SAW sensor housing (made of Kovar) and its FR4 support were computed in the same way. The obtained values are reported in the QUCS model presented in Figure 8. To take into account the pressure-dependent effect of the surrounding gas, an additional resistor must be used. The resistor models the energy leak into the gas. This resistance decreases when the gas pressure increases. It is known from [13] that the thermal conductivity of air in our present case can be approximated by λ = 0.592·S·P, where S is the gas-sensor surface contact area and P is the pressure in Pa. With S∼25.4 × 10^−6^ m^2^ (S is equal to the whole SAW sensor surface minus the bottom surface glued on the FR4 support minus the lateral surface in direct contact with the antenna) a sudden ±100 Pa (*i.e.*, ±1 mbar) pressure variation can therefore be modeled using a 665 Ω resistor. In addition, a programmable switch can be used to model pumping steps (see Figure 8).

In the thermal-electrical analogy, temperature corresponds to voltage and energy flow corresponds to current. Therefore, the heating energy can be directly included in the QUCS model by setting up a current source delivering one ampere (A) for one watt of heating energy. In our case, the heating energy was generated between the SAW sensor and its PCB support using a heating resistor connected to the Rx coil. The heating resistor is therefore the branching point between the Wireless Heating Model (WHM) from Section 3.1 and the Thermal Behavior Model (TBM) described above. Once both models are connected, it becomes possible to compute the behavior of the whole system, as follows. The current induced in the Rx coil must be first calculated using the WHM. The heating power generated by Joule effect in the heating resistor is then calculated and the equivalent current source in the TBM is set accordingly. Then, the QUCS model is run to compute the equivalent temperature behavior of the WiPirani. To get the temperature behavior of the SAW sensor, the voltage (*i.e.*, the equivalent temperature) must be taken right after the current source (see V_IN_ in Figure 8). Indeed, the heating resistor is directly in contact with the SAW sensor. To obtain the absolute temperature, it is also necessary to add the initial room temperature to the calculated V_IN_. Once the TBM and WHM are built and ready, the only required data to compute the WiPirani temperature behavior *versus* pressure are I_in_ (*i.e.*, the current injected in the Tx coil) and the distance between the two coils (D). The results obtained with I_in_= 60 mA and D = 15 mm are presented in Figure 4 (red line). There is a good agreement between experimental and numerical results.

### 3.3. Discussion

It can be seen from the QUCS equivalent circuit that the thermal resistance and thermal capacity of the antenna are big compared to the resistance and capacity of the other elements. It is therefore this building block that is mainly responsible for the very long response time of the whole WiPirani. The TBM-WHM model can be used to check what would be the response time of a WiPirani equipped with a much less massive antenna, made of free-standing aluminum wire (half-length = 6.2 cm, diameter = 0.1 mm). In this case, the equivalent resistance would be 1 kΩ and the equivalent capacity only 19 mF. For such an antenna, the TBM-WHM model yields a response time shorter than 60 s with a still good-enough sensitivity (0.12 °C/Pa). This would already constitute a significant improvement compared to what was obtained with the first WiPirani prototype. The response time might be further improved using miniaturization techniques. It will be the purpose of future work to use these techniques (including MEMS established solutions and processes) to design, fabricate and test a miniaturized and better performing version of the WiPirani. We think that an achievable target is a response time of 1 s, with an accuracy of ±0.1 Pa (±1.10^−3^ mbar).

## 4. Conclusions

We have demonstrated the technical feasibility of a fully wireless and passive low pressure sensor. The sensor is based on a SAW temperature sensor connected to a wireless induction-heating device and requested remotely using a specific Radar-like reader. The first prototype had a high sensitivity of 0.17 °C/Pa but a very long response time (τ > 5 min). A model was developed to account for the sensor's behavior. It was validated against measured data. Then, the model was used to test an improved configuration of the WiPirani using a much less massive antenna than the one used in the first demonstrator. It was shown that the response time might be easily reduced down to 40 s. Next steps will be the design, fabrication and test of a miniaturized version of the WiPirani. A response time of 1s with an accuracy of ±0.1 Pa are expected.
